# Fiber Optic Shape Sensing for Soft Robotics

**DOI:** 10.1089/soro.2018.0131

**Published:** 2019-10-04

**Authors:** Kevin C. Galloway, Yue Chen, Emily Templeton, Brian Rife, Isuru S. Godage, Eric J. Barth

**Affiliations:** ^1^ Department of Mechanical Engineering, Vanderbilt University, Nashville, Tennessee.; ^2^ Department of Mechanical Engineering, University of Arkansas, Fayetteville, Arkansas.; ^3^ Luna Innovations, Inc., Blacksburg, Virginia.; ^4^ School of Computing, DePaul University, Chicago, Illinois.

**Keywords:** fiber optic shape sensor, soft robotics, soft actuator, soft sensor

## Abstract

While soft material actuators can undergo large deformations to execute very complex motions, what is critically lacking in soft material robotic systems is the ability to collect high-resolution shape information for sophisticated functions such as environmental mapping, collision detection, and full state feedback control. This work explores the potential of a nearly commercial fiber optic shape sensor (FOSS) and presents the first demonstrations of a monolithic, multicore FOSS integrated into the structure of a fiber-reinforced soft actuator. In this pilot study, we report an open loop sensorized soft actuator capable of submillimeter position feedback that can detect the soft actuator's shape, environmental shapes, collision locations, and material stiffness properties.

## Introduction

Soft material robotics has captured the attention of academia,^1^ industry,^†^ and Hollywood.^‡^ Drawing from a highly compliant material library (i.e., elastomers and textiles), soft material robotics opens new avenues to create solutions that are closely matched to mechanical properties of natural and biological entities, thus enabling them to safely interface with everything from produce to people.^2–4^ Soft material robotic systems can execute large, complex elastic deformations with simple inputs such as pressurized fluid and cables and can apply small forces over large areas to safely perform supportive,^5,6^ rehabilitative,^3,4,7,8^ and manipulation^9–11^ functions. Many of the soft material actuators that have been proposed have an infinite degree of freedom of motion in their passive (unpowered) state and “preferred” degrees of freedom in their active state. This is very different from traditional rigid-robotic systems, which typically have finite configurations defined by the joint motions and are designed to transmit large forces with high precision. We emphasize preferred degrees of freedom because soft actuators have a continuum of “joint angles” with a subset of the continuum preferring motion under different circumstances. Consequently, external forces, collisions, and contact with obstacles can significantly alter the shape and motion of a soft actuator. To enable practical applications that can approach the precision, accuracy, and reliability of rigid robotic systems, advances in state feedback in soft material robotics systems are needed.

One research area that has emerged to address this challenge is soft sensors, which, in the context of soft material robotics, aim to minimally impede an actuator's range of motion while still providing sensory feedback such as proprioception,^12^ force/pressure sensing, strain, and curvature sensing.^13–16^ Resistive and capacitive soft sensors are among the most studied approaches, which combine electrically conductive materials with elastomeric and textile scaffolds. Motion is detected when the sensor is strained and the measured resistance or capacitance (depending on the configuration) changes. For example, there have been several demonstrations of these sensors detecting the movement of joints such as the knee, hip, elbow, and finger joints.^17–19^ While this category of soft sensors is adept at detecting motion at these high strain, single degree-of-freedom locations, there are considerable challenges to extending this technology to detect the three-dimensional (3D) shape of a soft material system along its continuum of joint angles. In addition, these sensors present challenges as a repeatable sensing method. Most notably, the hysteretic properties of the matrix elastomer and conductive material produce varying conductivities during cyclic loading.^13^

More recently, optoelectronic sensing has emerged as another soft sensor category where motion is detected through changes in the light that is emitted and received in a light guide.^8,20–22^ In particular, fiber optic intensity modulation (FOIM) is a common method that refers to a class of sensing techniques where light escapes from a light guide in response to some stimulus, such as bending of the optical fiber. Changes in intensity are then measured and associated with the stimulus. For example, Zhao *et al.*^8^ co-molded a single core etched plastic fiber optic cable into a soft bending actuator. In this particular design, the amount of light dissipation is affected by the amount of bending where changes in the amount of light detected on the receiving end of the cable can be correlated to a bending curvature. Since the length of the actuator is known, the curvature value can be used to infer and approximate the free deflection state of the actuator.

In another example, Sareh *et al.*^23^ used three macrobend sensors to measure the pose of a 40 mm long pneumatically actuated soft continuum arm. Each macrobend sensor consisted of a polymer optical fiber that had been sewn into an overlapping S-curve pattern along the length (i.e., up and back) of one side of the arm. The bend radii of the macrobend sensors would change in response to bending and extension of the arm, resulting in changes in the light intensity at the photodetector. In both of these examples, the FOIM technique can be used to detect motion and infer the actuator shape; however, this technique is limited by the assumption that the sensor curvature is uniform, since this technique can only measure the total light loss along the entire sensor. Consequently, this limits the ability of the sensor to accurately capture the actuator's pose when the actuator interacts with the physical world and deforms into nonconstant curvature configurations.

Recent work in optical sensing has also explored the design and integration of stretchable optical waveguides that use a lossy clear silicone core as the waveguide and correlate changes in transmitted light with elongation and bending deformation modes.^20^ The waveguides (1 mm diameter) are co-molded into the body of the actuator, enabling the sensor to seamlessly deform with the actuator. The authors demonstrate the ability to detect in-plane bending with a waveguide that loops longitudinally along the top half of the actuator where the actuator experiences the greatest strain. These were integrated into a soft prosthetic hand and demonstrated the ability to detect surface shapes and roughness. However, there are presently a few limitations of this sensing platform that must be considered early in the design process and in the context of the application. For example, the waveguide that is presented has anisotropic optical transmission properties due to the molding process. This enables the optical sensor to detect up and down bending; however, it cannot differentiate right from left side bending (and vice versa). Consequently, a single waveguide cannot accurately detect twist. To detect 3D motion, multiple sensors must be co-molded into the structure, which presents challenges with signal coupling and integration. Furthermore, while the stretchable core material can transmit light, the lossy nature of the material limits its sensing reach to lengths on the order of tens of centimeters and is not readily scalable to larger designs on the order of meters.

Finally, others have also explored optoelectronic shape detection with fiber Bragg gratings (FBGs), which reflect light with a peak wavelength that shifts in proportion to variations in strain and temperature.^24^ For example, the shape of continuum-type structures, such as in a biopsy needle^25,26^ and a cable-driven soft manipulator,^27^ have been measured by integrating multiple (three or more) FBG sensors circumferentially and parallel to the center axis of the structure. While this prior work has demonstrated 3D shape reconstruction, the proposed optical fiber arrangement is limiting. First, the proposed optical fiber arrangements were not able to detect the direction of twist along the center axis and, thus, assumed negligible torsional loading along the center axis during operation. For highly deformable systems,^10,28^ active and passive twisting are inevitable, and this optical fiber arrangement presents a significant source of error. Second, maintaining the precision spacing of multiple, independent optical fibers throughout a soft structure presents several manufacturing challenges, especially if the optical fiber is routed through a curve or along length scales on the order of meters.

While there have been considerable advances in the development of soft sensors, there remains a capability gap in soft robotic systems to accurately and precisely detect their own shape and their environment, which limits more advanced investigations into dynamic controls, collision detection, manipulation, locomotion, and material health monitoring, to name a few.

In this pilot study, we aim to provide a detailed record of how a nearly commercial monolithic, multicore, fiber optic shape sensor (FOSS) has the potential to address many of these challenges. Specifically, we demonstrate how an established soft actuator manufacturing process can be modified to incorporate a FOSS into the body of a fiber reinforced bending actuator. Moreover, through a series of experiments, we empirically demonstrate the capability of the actuator-sensor combination to capture in 3D space the actuator's shape, body twist, and tip position with submillimeter and subdegree resolution. To the authors' knowledge, this is the first demonstration of a FOSS integrated into a soft actuator that is capable of measuring bending and directionality of twisting motions. Finally, we demonstrate some of the capabilities of the actuator-sensor combination in a set of experiments that showcase its ability to detect a range of planar shapes, estimate contact locations along the actuator's length, and perceive the stiffness of surfaces selected from a range of soft materials.

## Materials

### Fiber optic shape sensor

**FIG. 1.**
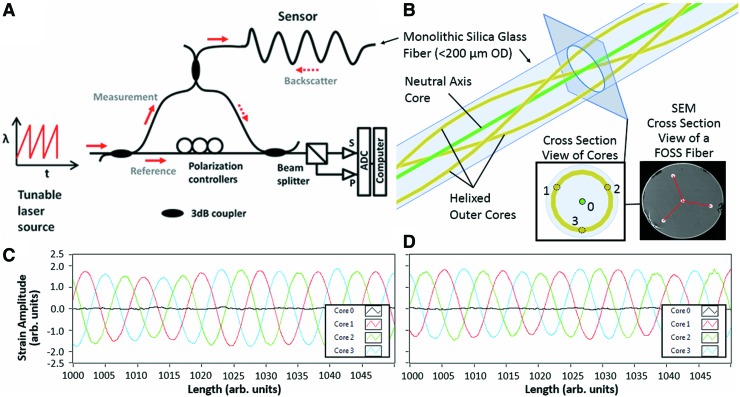
**(A)** Generalized OFDR optical network **(B)** Illustration of helical cores along a length of fiber with the inset image depicting the sensing triad—labeled 1, 2, and 3—and the core fiber—labeled 0. **(C)** Typical four-core strain response of the FOSS under pure bending, where the outer cores show sinusoidal strain centered around 0, while the center core remains neutral. **(D)** Typical four-core strain response of the FOSS under bending and twist, where the center core strain remains neutral, while the average of the outer cores is shifted upward or downward. FOSS, fiber optic shape sensing; OD, outside diameter; OFDR, optical frequency domain reflectometry; SEM, scanning electron microscope. Color images are available online.

In this pilot study, we use a research grade FOSS from Luna Innovations, Inc. (Blacksburg, VA). The operating principle underlying this FOSS platform is based on optical frequency domain reflectometry (OFDR), in which a laser is swept through a range of optical frequencies in a linear manner, producing interference fringes that arise from the recombination of light from reference and test paths,^29^ as shown in Figure 1A. A Fourier Transform is used to convert the interference fringes collected in the optical frequency domain at the S and P detectors into complex reflection coefficients in the optical time-of-flight delay domain. The resulting reflectivity versus distance plot has both very high spatial resolution (much less than 1 mm) and sufficient sensitivity to detect the Rayleigh backscatter that is reflected from random but permanent refractive features in the fiber's optical core. Comparing a scan of the optical core in a measurement state to a previously stored reference scan, minute changes in the local strain of the optical core can be detected. A more detailed discussion of the optics and mathematics that are used to make these high-definition, high-sensitivity OFDR measurements can be found in Kreger et al.^30^

3D position sensing is performed using OFDR techniques to monitor the distributed strain along the cores of a specialty multicore fiber. This fiber has four optical cores, one in the center and three outer cores that helix around the center core, as shown in Figure 1B. The cores are embedded into a single, monolithic glass fiber at the time of manufacture, with a helix rate of 66 turns per meter.^31^ The configuration of the multicore fiber can be uniquely determined by the strains along the cores: a region under curvature will show tension on the outer core on the outside of the bend and compression on the outer core on the inside of the bend. In practice, each of the outer cores experiences alternating states of tension and compression on its helical path through the bend. By comparing the amplitude and phase of these three strain curves (Fig. 1C), one can determine the applied bend radius (curvature) and its direction relative to the fiber's coordinate system. A region experiencing twist will show common-mode tension or compression on all outer cores, but not on the center core. By observing the magnitude of the common-mode strain signal, one can determine the distributed state of twist along the length of the sensing fiber. Strain measurements in the center core indicate axial strain or temperature change in the shape sensing fiber. The local measurements of curvature, twist, and axial strain are then used to calculate the Cartesian X, Y, Z position of the sensing fiber in 3D space.^31^

There are several features worth noting about this multicore FOSS platform. First, the fiber optic cable is compliant with a bending radius of curvature as low as 10 mm and can maintain functionality under significant morphological variation. Second, the FOSS can detect bending and twisting along the entire length of the sensor with sensor measurements every 0.8 mm, thus enabling high fidelity full 3D state estimation with sampling rates up to 250 Hz (see Supplementary Data and Supplementary Table S1 for more information). It should be emphasized that the shape measurement is a measurement of the path of the fiber in 3D space and does not require knowledge of the mechanical properties (e.g., stiffness) of the structure it is monitoring. In this work, our study was conducted with a 1.35 m long FOSS; however, the sensor length is highly scalable and can operate at tens of meters (though at lower sampling rates). Third, the sensor's cross-section diameter (∼200 μm) enables it to be configured into a range of elastomeric, textile, and other compliant structures. Fourth, the FOSS can be integrated into structures, thus eliminating the need for line of sight. Fifth, this particular platform by Luna Innovations does not require a detector at the end of the sensor. Therefore, the sensor can terminate within a structure and not circle back to a detector point, thus maximizing its spatial reach.

While there are several notable advantages to this FOSS system, it should be noted that the FOSS is not stretchable; therefore, consideration early in the design must be given to its placement such as along a neutral axis, lines of nonextension, or configuring the sensor to uncoil as the soft structure deforms. In addition, even though the sensor itself has a very low profile, the enclosure housing the supporting hardware (i.e., laser, optics, and electronics) is much larger, with a footprint near that of an A5 sheet of paper by 15 cm tall. This presents challenges for some mobile applications where carrying capacity is limited and ruggedness is essential.

### Fiber-reinforced soft actuator with integrated FOSS

**FIG. 2.**
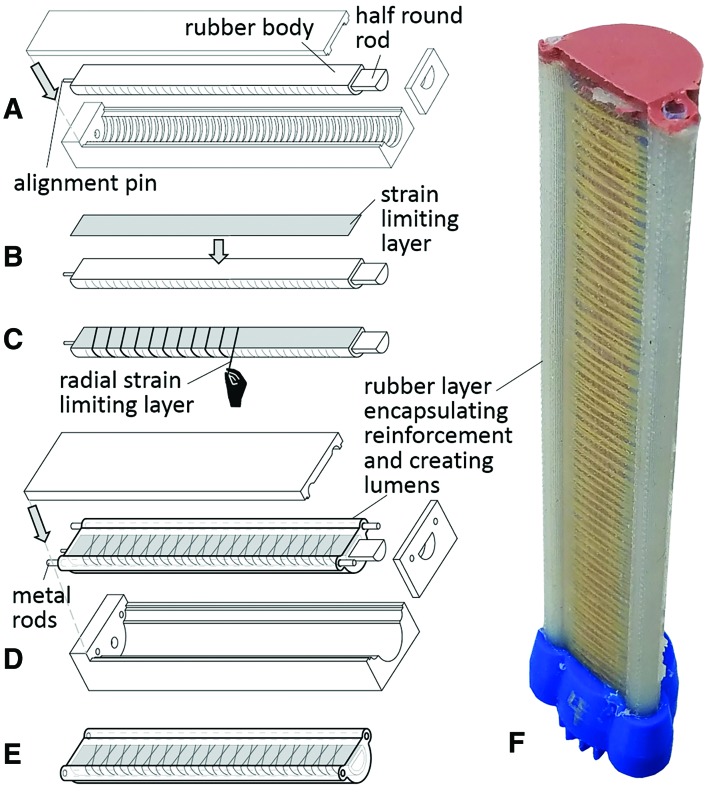
Schematic diagram highlighting the fabrication process for a soft fiber-reinforced bending actuator with integrated lumens. **(A)** The first molding step using a 3D printed three-part mold to define the exterior shape of the rubber body around a half-round steel rod. **(B)** The strain limiting layer (woven fiberglass) is attached to the flat face of the actuator. **(C)** Fiber reinforcements (Kevlar fiber) are wound along the entire length of the actuator. **(D)** The second molding step, the entire actuator is encapsulated in a 2.0 mm thick layer of silicone to anchor all fiber reinforcements and to create lumens with the 3.175 mm diameter steel rods that run parallel to the long axis of the half round rod. **(E)** All the steel rods are removed and both ends of the actuator are capped with a connection point on one end for pressurized fluid. **(F)** Fabricated soft actuator measuring 16 cm in length. 3D, three-dimensional. Color images are available online.

In prior work,^4^ a multistep soft actuator manufacturing method was presented for molding elastomeric tubular bladders whereby fiber reinforcements can be embedded in the actuator wall to influence the material's strain response to a pressurized fluid input. We present an enhancement to this manufacturing method that co-molds lumens lined with Teflon tubing to support the installation and removal of the FOSS. As shown in Figure 2, the first three steps of the fabrication process define the shape of the actuator's bladder and the fiber reinforcements. In the fourth step, we present a new feature by co-molding other structures in addition to encapsulating the fiber reinforcements. For the purpose of this work, we needed a method to integrate the FOSS along the perimeter of the actuator. The FOSS sensor comes housed in a 3-mm outside diameter furcation tube (part no.: F00FR3NUY; Fiber Instrument Sales, Inc., Oriskany, NY) that is intended to protect the sensor from bending beyond its 10 mm radius of curvature. The furcation tube also provides a low friction housing for the FOSS to slide in to prevent the sensor from experiencing potentially damaging compressive and tensile loads during handling. Given the protective and functional utility of the furcation tube, the soft actuator was designed to enable manual installation of the FOSS assembly. In this fourth step, the mold holds two 3.175 mm diameter steel rods placed on either side of the fiber-reinforced actuator and coplanar with the strain limiting layer (i.e., the neutral axis). Thin-walled Teflon tubes were cut to length and placed over the steel rods. The mold was assembled without the cap, filled with polymer (Dragon Skin 20 by Smooth-On, Inc.), and degassed in a vacuum chamber. The mold was then capped to ensure alignment of the actuator and steel rods and placed in a pressure chamber at 70 psi to fully cure. The cured actuator was then removed from the mold, and one end was attached to a pneumatic fitting and the other end was capped by placing the open end in a 10 mm deep container of mixed silicone (M4601 by Wacker Chemical) and allowed to cure overnight. See Figure 2F for an image of a completed actuator.

### Actuator and sensor integration

In the present design, the FOSS and furcation tube pass through the base of the actuator through one lumen and then loop around the end to return through the other lumen. A 3D printed end cap clamps to the end of the actuator and holds the curved section of the sensor at a fixed radius—12 mm. The specific arrangement was selected for three reasons. First, positioning the sensor along the neutral axis (i.e., the strain limiting layer) on either side of the actuator enables detection of any active or passive twist in the body of the actuator. Second, constraining the curved portion of the sensor at the distal end of the actuator in a plastic cap serves as a registration technique to identify the end of the actuator. Since the FOSS is allowed to slide freely inside the furcation tube, there is the potential for the position data measured by the sensor to shift relative to the end of the actuator. The fixed shape of the curve section serves as a geometric locator where the point of the curve of the sensor that is furthest from the base of the actuator can be assumed to be the furthest point measured on the actuator. Finally, constraining the curved portion of the sensor to a plane enables techniques to extend the reach of the sensor and customize it for specific applications. For example, in the present example the cap has a narrow paddle shape that extends beyond the end of the actuator and permits access into tighter spaces. If we assume that the FOSS and end cap are rigidly connected, then we can take the known geometry of the cap along with the position and orientation of this curved portion and calculate the location and orientation of the end of the cap.

**FIG. 3.**
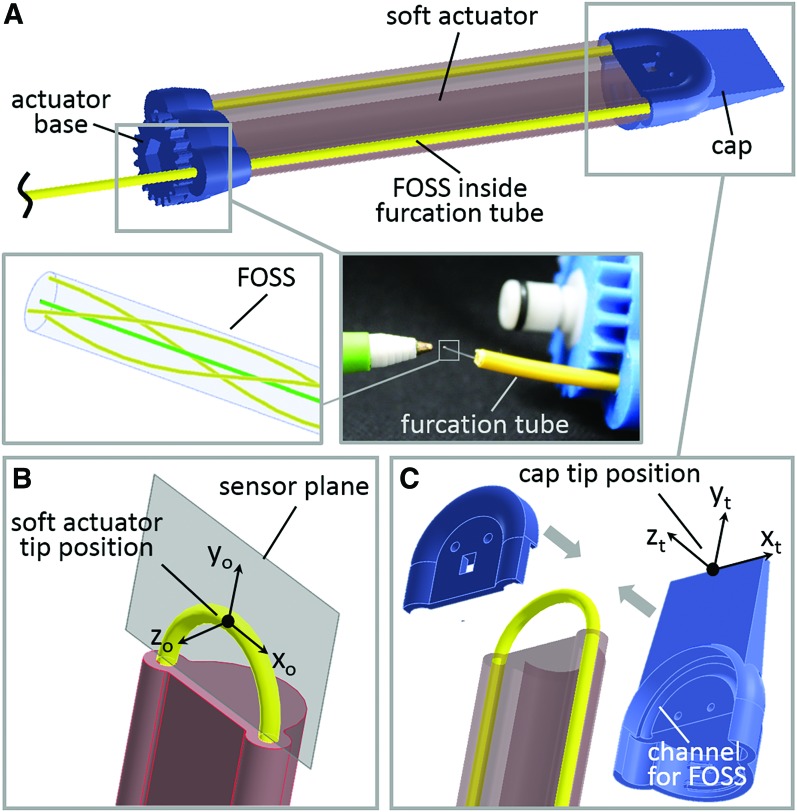
**(A)** Path of the FOSS when it is passed through the actuator's lumens. **(B)** U-shape of the sensor as loops around the end to return through the other lumen. We assume that the center line of the FOSS lies in a plane—sensor plane. **(C)** Exploded view of the rigid plastic cap that is attached to the end of the sensor to reinforce the U-shape in the sensor plane. Color images are available online.

More specifically, the robot tip can be identified as the point where the *z*-directional gradient reaches the maximum value. Using the known geometry of the cap, the segment of the FOSS within the cap is approximated to be a planar curve due to the restriction of the predefined semicircular slot in the cap—labeled *channel for FOSS* in Figure 3C. The soft actuator tip coordinate frame (*x*_0_, *y*_0_, *z*_0_) can be derived using the singular value decomposition (svd) method^32^ of the point matrix (*A*) of the FOSS segment inside the cap.

\begin{align*}
\left[ {U , S , V} \right] = svd \left( A \right) \tag{1}
\end{align*}

The normal vector of the plane defined by the semicircular slot in the cap can be found at the third column of the *V* matrix in the local coordinate frame shown in Figure 3B, which can be written as

\begin{align*}
\textbf{\textit{N}} = \textbf{\textit{V}} \left( {: \; , \textbf{\textit{end}}} \right) \tag{2}
\end{align*}

Since the binormal vector is $\textbf{\textit{B}} = { \left[ {0 , 0 , 1} \right] ^T}$, the tangential vector $\textbf{\textit{T}}$ can be calculated according to the right hand rule, which is the cross product of the binormal vector and normal vector. Using the known geometry of the cap, the cap tip position (Fig. 3C) can be calculated through the rigid body transformations, which can be written as

**FIG. 4.**
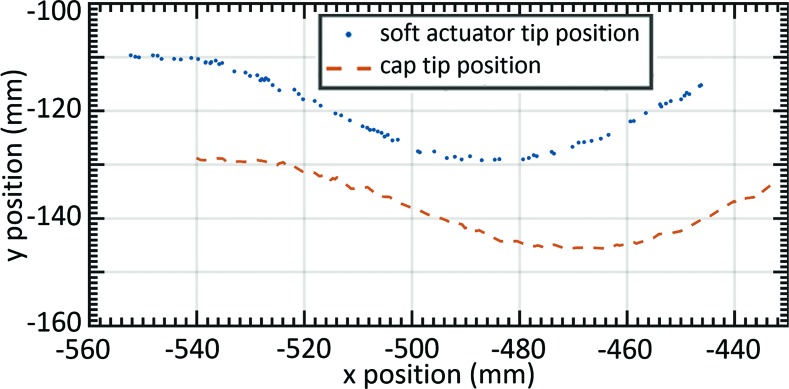
Soft robot tip position and cap position as the tip of the actuator is dragged along a surface of a sinusoid shape. Color images are available online.

\begin{align*}
{P_{cap}} = {P_{robot}} + a\textbf{\textit{N}} + b\textbf{\textit{T}} , \tag{3}
\end{align*}

where *a* and *b* are the offset in the normal and tangential direction, respectively. Figure 4 demonstrates the rigid transformation method where the known robot tip position can be correlated with the corresponding cap tip position (*x*_t_, *y*_t_, *z*_t_). For the remainder of this article, when we refer to *tip position* or *tip orientation*, we refer to these data after the rigid transformation or cap tip position and orientation.

## Experimental Methods

The following subsections detail several experimental setups designed to characterize the FOSS's accuracy, as well as the ability of the actuator-sensor combination to detect obstacles, material stiffness properties, and planar shapes. All the experiments were conducted at the Luna Innovations research facility (Blacksburg, VA) with their FOSS evaluation platform. Shape data were collected on a Luna operated computer, while all other data (e.g., soft actuator linear translation motion, supplied compressed air, load cell, and pneumatic valve control signal) were collected on a MATLAB Simulink Real-time Target machine at the sampling rate of 1 kHz. Given the early stage nature of this work, the experiments are all open loop.

In all the experiments, the FOSS was fed through one side of the actuator and then down the other side, as illustrated in Figure 3A. This placed P_robot_ ∼1.25 m from the FOSS origin. This is important to note because position data error does build as the distance from the FOSS origin increases. For example, in the stationary experiments below (i.e., where the actuator base does not move), more of the FOSS could have been fed through the actuator to shorten the length of the FOSS between its origin and P_robot_ and, thus, produce data with less error. We chose a distal location on the FOSS to characterize its spatial reach where the error is most significant.

### Tip twist experiment

**FIG. 5.**
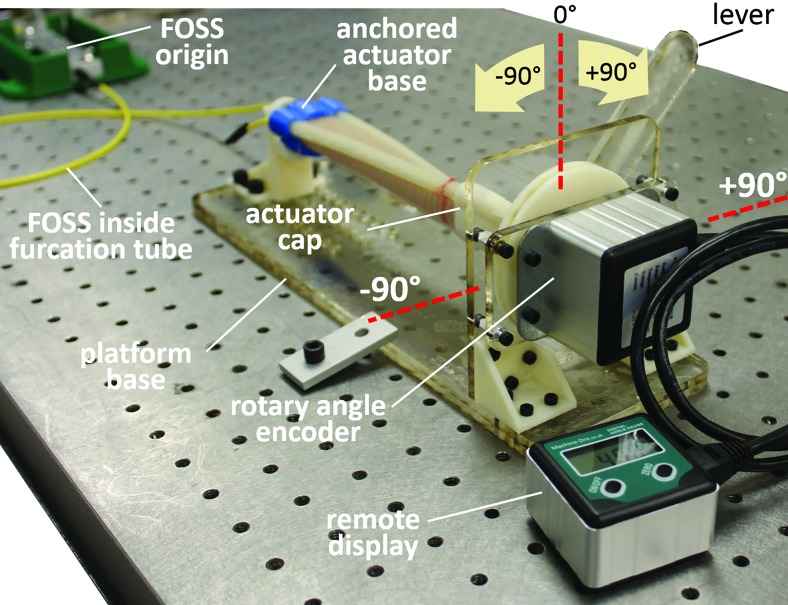
Experimental setup for measuring the twist at the tip of the actuator, while the base of the actuator is anchored. The tip of the actuator was rotated in increments of 5° to a maximum of −90° and +90°. Color images are available online.

In the actuator tip twist experiment, we examined the ability of the FOSS to measure twist along the actuator's longitudinal axis. The experiment consists of a platform that rigidly fixes the base of the actuator, and attaches the actuator cap (i.e. actuator tip) to a disc that only permits rotation about the actuator's longitudinal axis (Fig. 5). A rotary angle encoder (part no.: ME-AN-REM-ROT by Machine DRO) with 0.1° resolution measures the magnitude of twist relative to the actuator base. A lever extending from the disc was used to manually control the amount of rotation at the tip of the actuator. The zero position of the lever arm was set with a carpenter's square to position the lever perpendicular to the platform base. Using the digital remote display, the disc and ultimately the actuator tip orientation were rotated in increments of 5° to a maximum of 90° in the clockwise and counterclockwise directions. At each increment, shape data from the FOSS were recorded. The tip orientation angle $\theta$ was calculated using the following equation

\begin{align*}
\theta = atan2 \left( {{N_1} , \;{N_2}} \right) , \tag{4}
\end{align*}

where *N*_1_ and *N*_2_ are the normal vectors of the tip section and the base section, respectively. The normal vectors can be obtained according to the method described in Equations (1) and (2). It should be noted that *N*_2_ is a constant vector since the base of the actuator is rigidly fixed (Fig. 5).

### Actuator tip tracking: linear translation

**FIG. 6.**
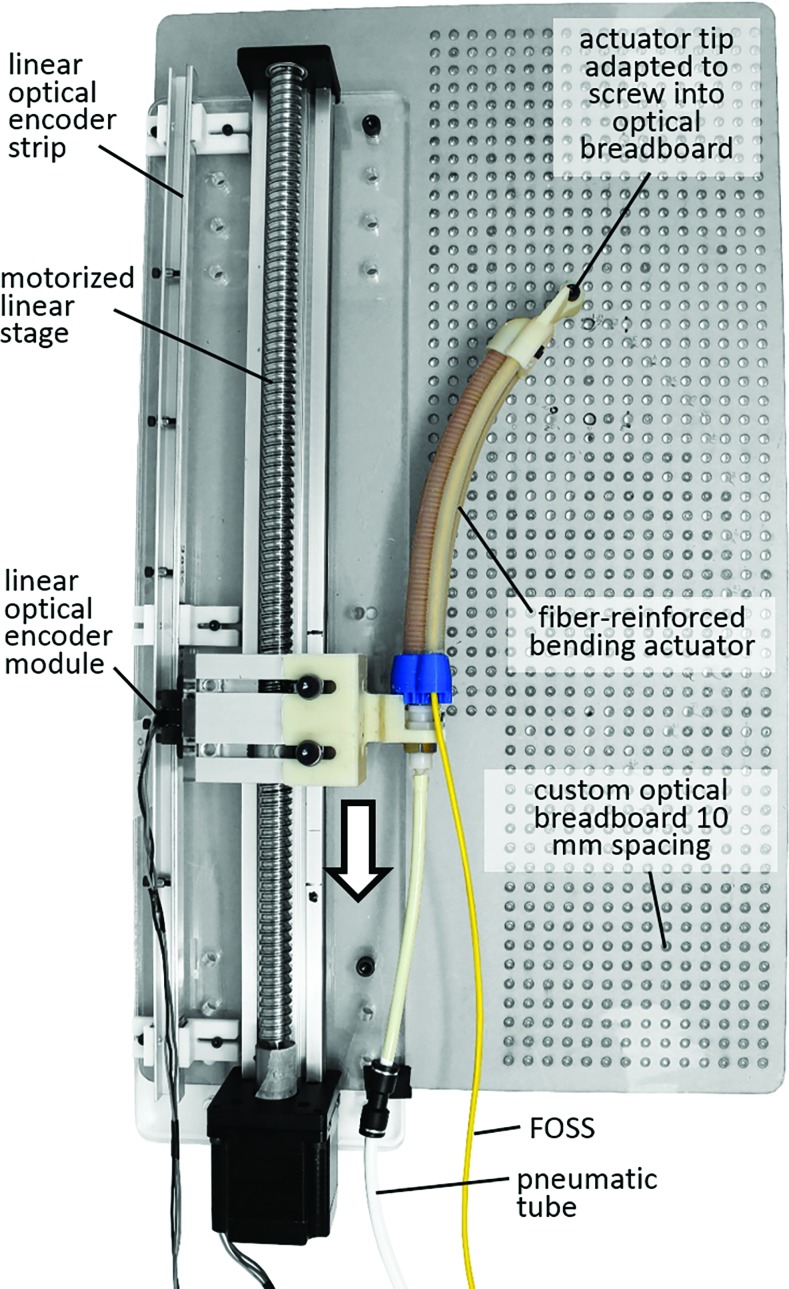
Top view of the platform used for the linear translation, contact detection, and shape detection experiments. Color images are available online.

The linear translation, contact detection, and shape detection experiments were all conducted on a common platform (Fig. 6) that consisted of a motorized linear stage (part no.: 200180 by Konmison) capable of 400 mm of linear travel at 50 mm/s, a 20-inch (508 mm) linear encoder (optical encoder module part no.: EM2-0-2000-I; linear strip part no.: LIN-2000-20-1 from US Digital, Vancouver, WA) with 2000 counts per inch for recording the linear stage position, and a custom laser cut acrylic optical breadboard with 10 mm hole spacing. The encoder data were processed through a 32-bit pulse counter (Contec CNT32-8M). In all the experiments, actuator pressure was recorded with a Festo pressure sensor (SPTW-P10R-G14-VD-M12) and controlled with a Festo valve (MPYE5-M5-010-B).

**FIG. 7.**
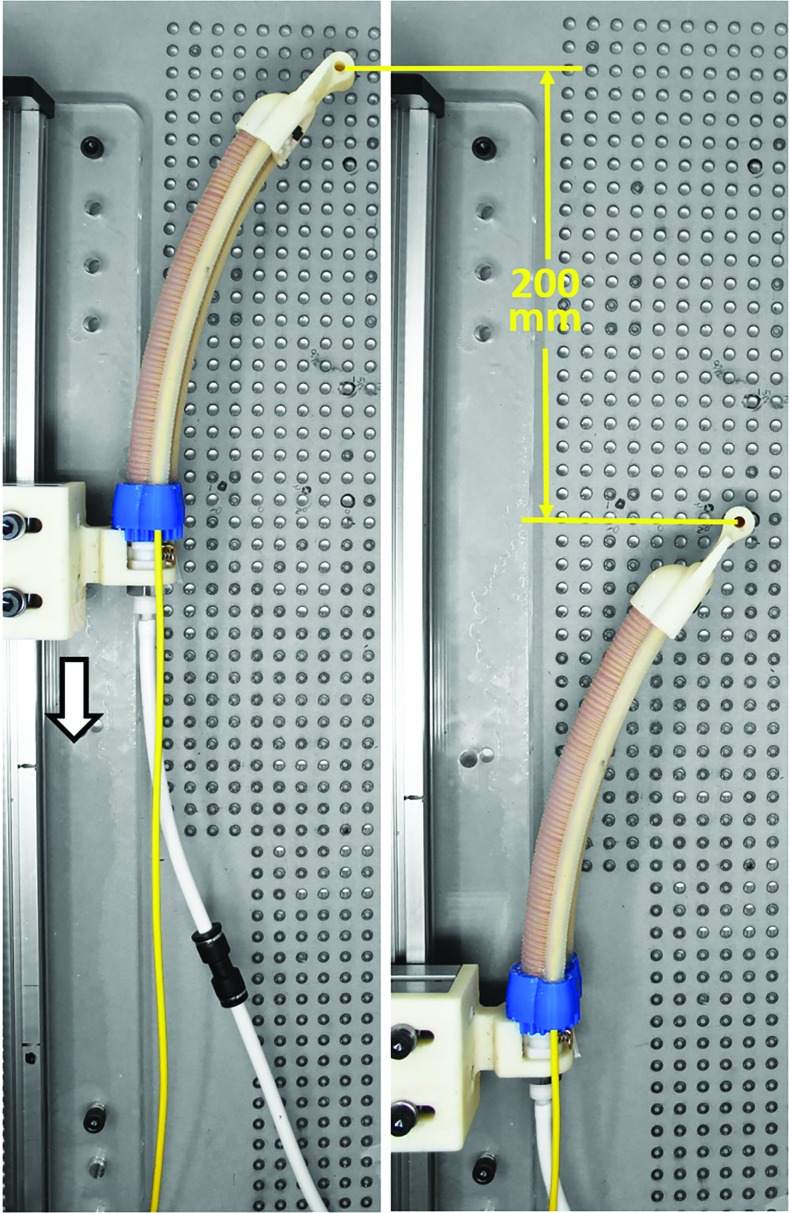
Experimental setup for tracking the tip of the actuator when the actuator-sensor combination was moved 200 mm along a linear path on a motorized linear stage. Color images are available online.

In the linear translation experiment, we evaluated the accuracy of the shape sensor to record the tip position of the actuator as it moved along a linear path. Compared to the twist experiment, where the FOSS leading up to the actuator remained stationary, in the linear travel experiment the entire FOSS moves. The purpose of this experiment was to evaluate the positional tip accuracy of the FOSS with a known trajectory. The actuator base was rigidly attached to the linear stage and driven 200 mm at 50 mm/s (Fig. 7). The actuator was not pressurized during this experiment. The experiment was repeated five times.

### Actuator tip tracking: bending actuator experiment

**FIG. 8.**
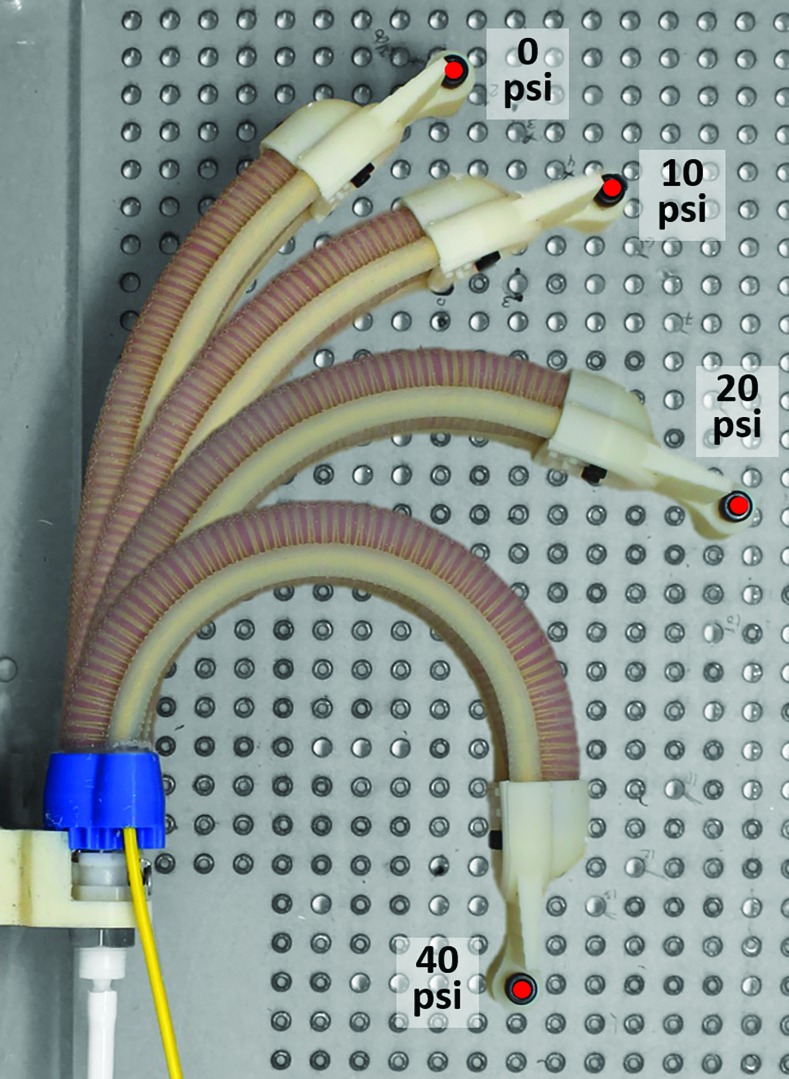
Overlays of the actuator-sensor combination at different pressures, illustrating the experimental setup for tracking the tip of the actuator at multiple points on the optical breadboard that lie closest to its free deflection path. The optical breadboard served as the ground truth for evaluating the FOSS data. Color images are available online.

Under free deflection conditions (e.g., no obstacles), the proposed soft actuator bends primarily along a two-dimensional (2D) plane as it is pressurized. In this experiment, we characterized the ability of the FOSS to accurately capture the tip position of the actuator at several different pressures, namely 0, 2.5, 5, 7.5, 10, 12.5, 15, 17.5, 20, 25, 30, 35, and 40 psi. The proximal end of the actuator was fixed to the linear stage. At each pressure increment, the tip of the cap was secured to the nearest breadboard hole. Due to limited access to alternative motion tracking tools at the test facility, the custom acrylic optical breadboard served as the ground truth for this experiment. Therefore, while the tip positions presented in this study do not reflect true, free deflection conditions, they are representative of the range of motion of the actuator and demonstrate the capability of the FOSS to operate under these loading conditions. Figure 8 presents a top view of the experiment with overlays of the actuator-sensor combination at several states of pressurization.

### Collision detection

**FIG. 9.**
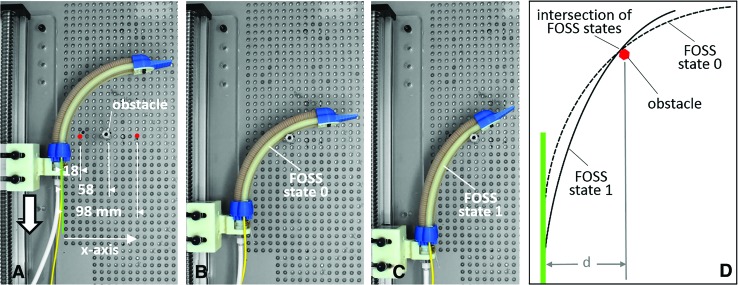
**(A)** Top view of the collision detection experiment with the location of the aluminum standoff obstacle at 18, 58, and 98 mm relative to the base of the actuator. **(B)** Depicts the instant the soft actuator contacts the obstacle. The shape of the sensor is recorded as FOSS state 0. **(C)** Depicts the deformation of the actuator-sensor combination some distance further down the linear stage. The shape of the sensor is recorded as state 1. **(D)** Diagram of the shape data from states 0 and 1 overlaid to identify their intersection and thus the location of the obstacle. Color images are available online.

The proposed 2D collision detection experiment explores a unique and straightforward capability of the shape sensor to detect the location of a collision anywhere along the length of the actuator's inside face. Using the same custom optical breadboard as the ground truth, an aluminum standoff (i.e., the obstacle) was anchored at three different points along the *x*-axis (Fig. 9), namely 18, 58, and 98 mm from the base of the actuator. The actuator was pressurized to 15 psi, and the linear stage moved the actuator-sensor combination (Fig. 9A) to collide with the aluminum standoff (Fig. 9B). At the point of collision—labeled FOSS state 0 in Figure 9B and D—there is a detectable change in the shape of the actuator. As the actuator is driven further down, the change in the shape of the actuator grows, as indicated in the example of FOSS state 1 in Figure 9C and D. By overlaying the shape scans of the actuator between state 0 and state 1, the intersections of the shape data were used to estimate the location of the standoff.

### Planar mapping

**FIG. 10.**
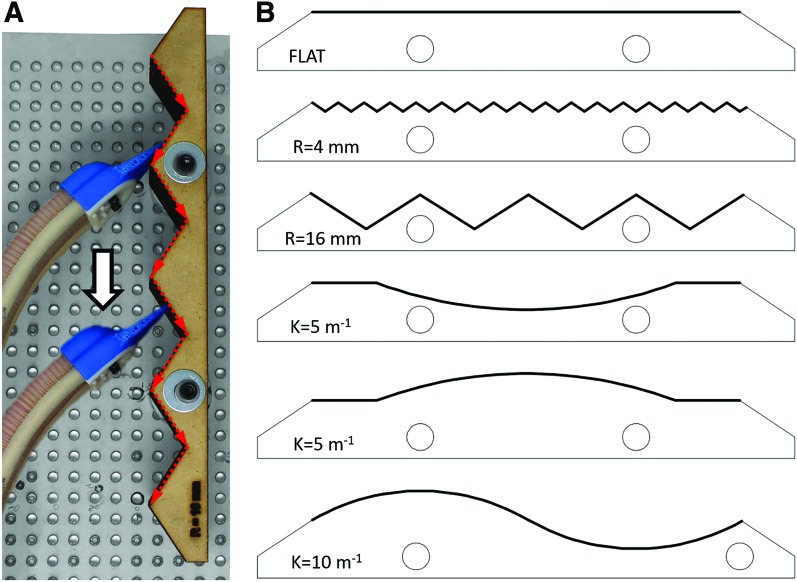
**(A)** Top view of the actuator tip sweeping over a 16 mm amplitude sawtooth surface that was anchored to the optical table. It should be noted that the red arrows highlight the path of the actuator tip as it sweeps over a surface. **(B)** Overview of all the surface geometries that were evaluated. Color images are available online.

In the planar mapping experiment, we evaluated the capability of the actuator-sensor combination to accurately capture the shape of a surface as the actuator tip swept over it. The surfaces that were evaluated include a flat, 4 mm amplitude sawtooth, a 16 mm amplitude sawtooth, a convex curve, a concave curve, and a sinusoid shape (Fig. 10). These surfaces were inspired by similar surfaces presented in Zhao et al.^20^ The shapes were constructed from 7 layers of 6.15 mm thick laser cut medium density fiberboard. The layers were fixed to the custom optical breadboard, and the cap tip swept over the surfaces at 50 mm/s on the motorized linear stage. We found that the actuator made sufficient contact with all the evaluated surfaces when it was pressurized to 15 psi. The FOSS shape data were stored in the shape sensor coordinate frame with an update rate of 75 Hz. The robot tip position was identified according to the method presented in the Actuator and Sensor Integration section. The 2D shapes were approximated by the tip trajectory, since the actuator tip was in contact with the objects during the test. To validate the shape-mapping accuracy, the robot tip trajectory data in the shape sensor coordinate frame were registered to the ideal shape through the iterative closest point algorithm.^33^

### Material stiffness experiment

**FIG. 11.**
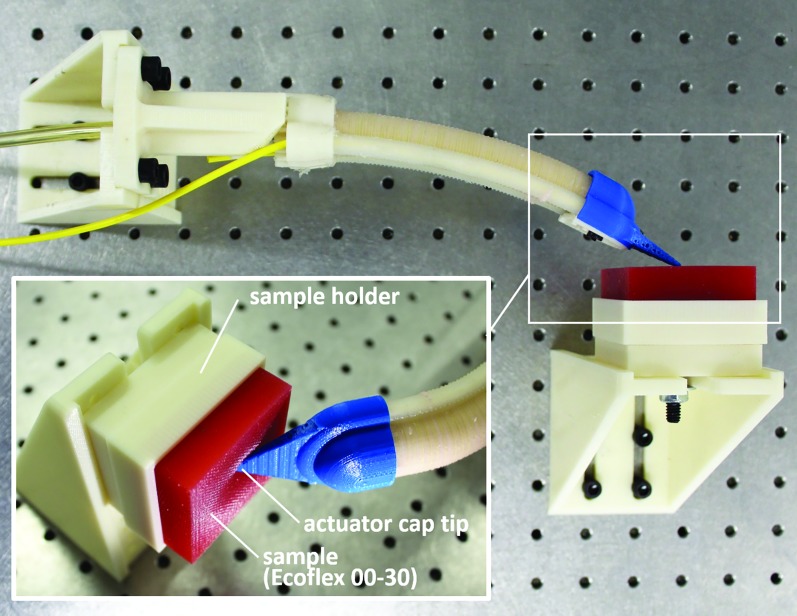
Top view of the actuator-sensor combination anchored to an optical table using right angle mounts with the tip of the actuator resting on the surface of a test sample. The inset image shows a close-up of the actuator pressurized to 276 kPa (40 psi) and the tip pressing into the sample material. Color images are available online.

In the last experiment of this work, we examined the capability of the actuator-sensor combination to distinguish the relative stiffness of several materials, including a rigid material (acrylic), 50A durometer silicone (SilPro 950 by Reynolds Advanced Materials), 20A durometer silicone (Dragon Skin 20 by Smooth-On, Inc.), 00-30 durometer silicone (Ecoflex 00-30 by Smooth-On, Inc.), and ultrasoft memory foam, which is designed to compress 25% with 0.2 psi (part no.: 86195K35; McMaster-Carr, Inc.). All samples measured 50 mm (W) × 50 mm (D) × 19 mm (H) and were placed in a sample holder. With an initial pressure of 0 psi, the actuator was positioned where the cap tip barely touched the sample material (Fig. 11). The shape of the actuator and, ultimately, the position of the tip were recorded by the FOSS as the actuator was pressurized to 276 kPa (40 psi). The distance the tip of the actuator indented into the sample material was used as the measure of the stiffness of the material. It should be noted that to improve the stiffness testing capabilities of the actuator-sensor combination, the actuator was shortened to 11 cm from 16 cm. In a benchtop test, we found that this increased the tip force at 276 kPa by 31% to 4.5 N from 3.4 N. Furthermore, the cap tip shape was modified to narrow to a 4 mm wide edge, down from a 25 mm edge, to concentrate the tip force into a smaller zone and improve the depth of material indentation.

## Results

### Tip twist experiment

**FIG. 12.**
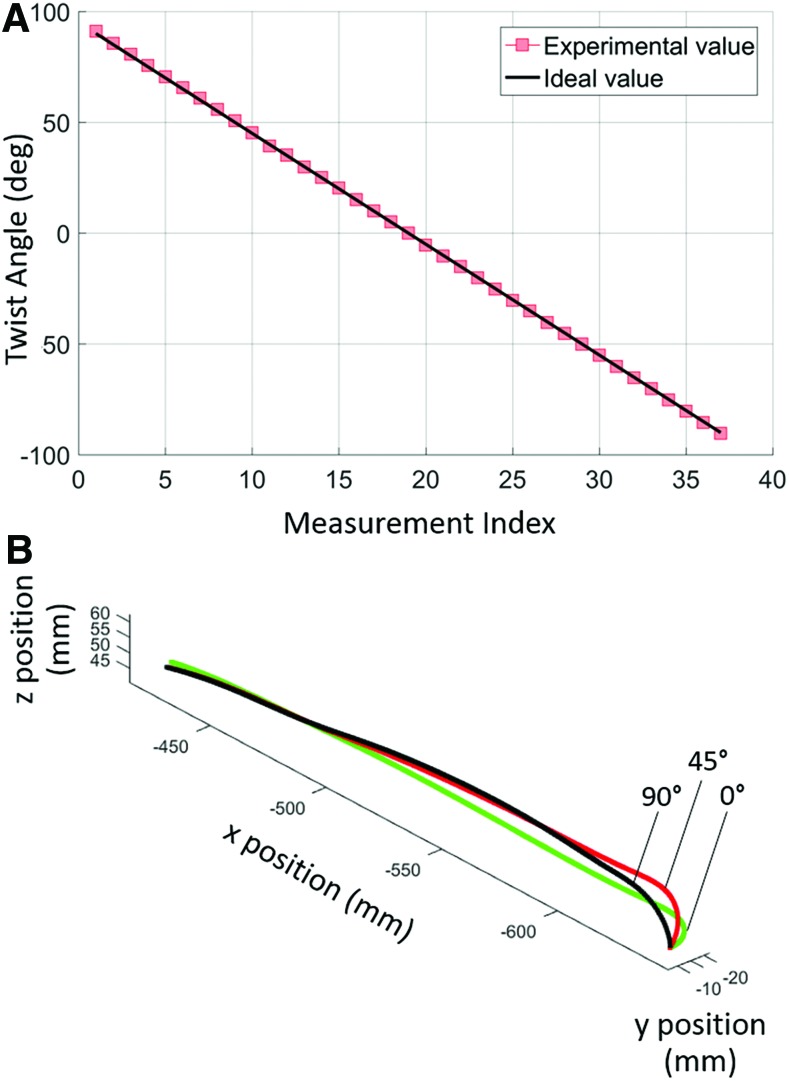
**(A)** Tip angle measured by the FOSS as it was rotated in 5° increments from +90° to −90°. **(B)** 3D view of the FOSS shape data at three different twist positions—0°, 45°, and 90°. Color images are available online.

Figure 12A presents a plot of the tip angle measured by the FOSS and the rotary angle encoder as it was rotated in 5° increments from +90° to −90°. Furthermore, Figure 12B plots the shape data in a Cartesian coordinate frame at three different twist angles, namely 0°, 45°, and 90°. It should be noted that to minimize confusion in the presentation of the shape data in Figure 12B, only the shape data up to the apex of the cap tip are presented. Across the 37 measurements in this experiment, the FOSS had a mean error of 0.37° and a standard deviation (SD) of 0.26 (see Table 1 for a summary of results).

**Table 1.** Summary of Experimental Results

*Experiment*	Mean error (mm)^*^	*SD*
Accuracy test		
Tip twist (°)	0.37	0.26
Linear translation	0.13	0.09
Bending	0.64	0.48
Contact detection		
18 mm offset	1.3	0.5
58 mm offset	2.3	1.2
98 mm offset	2.7	1.7
Planar mapping		
Flat	0.29	0.19
4 mm sawtooth	0.98	0.62
16 mm sawtooth	0.99	0.60
5^−1^ m concave	0.91	0.61
5^−1^ m convex	0.90	0.64
10^−1^ m sine wave	0.70	0.59

^*^ *Note:* The tip twist experimental results are expressed in degrees.

SD, standard deviation.

### Actuator tip tracking: linear translation

**FIG. 13.**
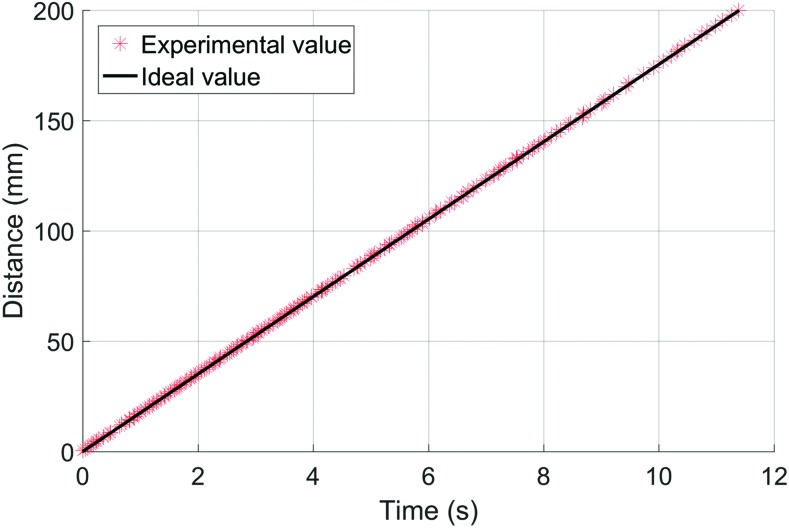
Tip position of the soft actuator, which is 1.25 m from the FOSS origin, as the actuator traversed 200 mm along the motorized linear stage. Color images are available online.

In this one-dimensional experiment, Figure 13 plots the tip position of the soft actuator as the actuator traversed 200 mm on the motorized linear stage. The FOSS data compared to the linear optical encoder had a mean error of 0.13 mm and a SD of 0.09 (Table 1). We should note that this motion study, as opposed to the static measurement of the tip twist experiment, highlights a data limitation of the FOSS evaluation platform. The FOSS system outputs time-stamped data at 250 Hz; however, the computer saving the data could only store it at a rate up to 75 Hz, and even then there are time periods where it dropped lower than this due to limitations of the computer's operating system. This is evident in Figure 13, where the density of the experimental results decreases noticeably about halfway through the experiment until the end. This is a limitation of data collection system and not of the actual FOSS.

### Actuator tip tracking: bending actuator experiment

**FIG. 14.**
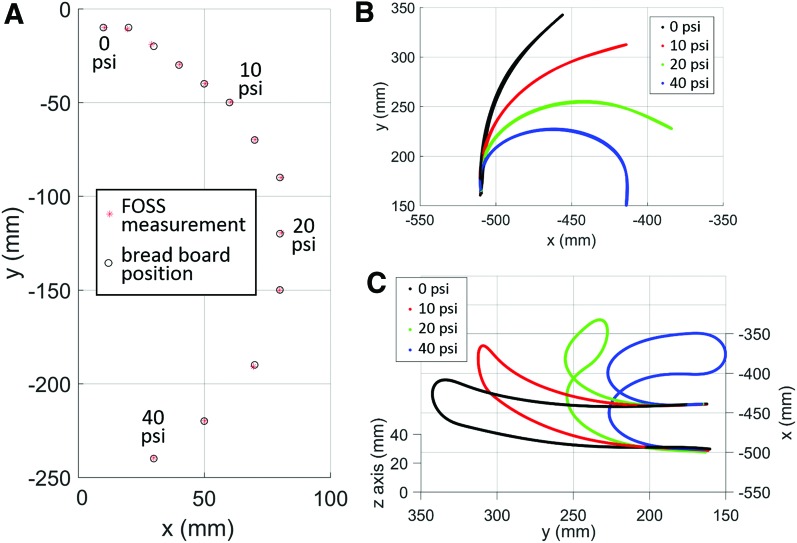
**(A)** Tip position of the soft actuator as measured by the FOSS compared to the known anchor points in the optical breadboard. **(B)** Side view and **(C)** 3D view of the shape data at several different pressures—0, 10, 20, and 40 psi. Color images are available online.

In the bending actuator experiment, the FOSS demonstrated a similar submillimeter mean error—0.64 mm—as the tip of the actuator was registered to more than a dozen points on the custom optical breadboard. Figure 14A plots the FOSS measurements and the breadboard positions at the different actuation pressures. It should be noted again that these measurement values are collected 1.25 m from the FOSS origin. Hence, we are able to conclude that under these static conditions the sensor can safety operate inside the soft actuator across large deflections and without significant loss of measurement accuracy. In Figures 14B and C, a side view and 3D view, respectively, of the shape data are presented at 0, 10, 20, and 40 psi. For clarity, we only plotted the shape data of the FOSS, that were fully within the actuator. It is worth noting that the FOSS and the furcation tubing are very flexible and do not significantly alter the range of motion of the assembled soft actuator. This was verified in a simple experiment where the actuator was pressurized to 40 psi with and without the sensor assembly (i.e., the FOSS and furcation tubing). The difference in the cap tip position was less than 6 mm, representing a reduction of less than approximately 2.3% in the range of motion.

### Collision detection

For the collision detection experiment, the actuator-sensor combination was capable of detecting the obstacle position at 18, 58, and 98 mm from the actuator base with an average error of 1.3, 2.3, and 2.7 mm across three experiments (see Table 1 for a summary of results), with a 3.8 cm travel of the linear stage. This is a topic that requires further exploration, which is beyond the scope of this article, to improve the accuracy of obstacle position detection. With respect to these first results, we observed that the error in position detection increased as the obstacle was located more distal to the actuator. This is in one respect an inherent limitation of the sensor, where error in the position data increases the further the reading is from the FOSS origin. We also suspect that this is due to the lever-like contact between the soft actuator and the obstacle. When the obstacle is near the base of the actuator (i.e., 18 mm away), this creates a small lever arm. Therefore, small advancements of the linear stage produce substantial changes in the shape of the actuator (i.e., state 0 vs. state 1) extending past the obstacle. When the obstacle is further away, the actuator does not deform as much for the same linear travel, which leaves the potential for more error in detecting the obstacle location.

### Planar mapping

**FIG. 15.**
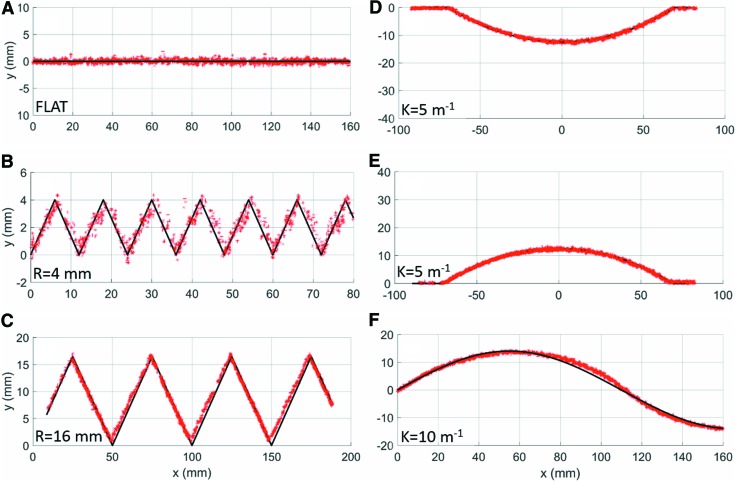
Experimental results of the actuator-sensor combination to inspect and reconstruct six different planar shapes, with submillimeter error: **(A)** flat, **(B)** 4 mm amplitude sawtooth, **(C)** 16 mm amplitude sawtooth, **(D)** convex curve, **(E)** concave curve, **(F)** sinusoid. Color images are available online.

In Figure 15, we present the capability of the actuator-sensor combination to reconstruct six different surfaces with a submillimeter average error. It should be noted that as part of this study, smaller sawtooth surfaces—0.5 and 1 mm amplitudes—were evaluated; however, there was too much noise in the data to be able to identify the surface features. For our particular setup, the spatial data collected at 1.25 m from the FOSS origin are going to have more noise compared to spatial data collected closer to the origin. Thus, it is important to consider the placement of the FOSS and the resulting sensor resolution early in the design process.

### Material stiffness experiment

**FIG. 16.**
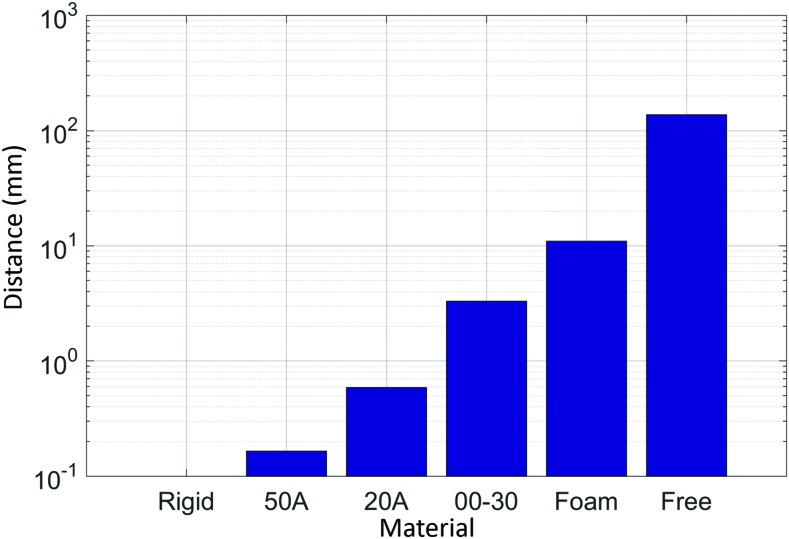
Experimental results of the actuator-sensor combination to detect the relative difference in stiffness across a range of compliant materials. Color images are available online.

In our final experiment, we present the results of the actuator-sensor combination to detect the stiffness of several different materials (Fig. 16). The measured actuator tip indentations into these materials were normalized against the acrylic. The results suggest that the system is capable of detecting the relative stiffness of materials for durometers 50A and softer. It should be noted that it is feasible to detect stiffer materials by increasing the sensor accuracy (i.e., reducing the distance between P_robot_ and the FOSS origin), and by increasing the actuator tip force.

## Discussion

In this article, we presented a design and characterization study that integrates a FOSS into the body of a soft fiber-reinforced bending actuator to provide details about the shape and the tip position of the actuator. In our set of accuracy tests—tip twist, linear translation, and bending—the sensor was capable of capturing the degree of twist and tip position with subdegree and submillimeter precision (Table 1). These initial constrained demonstrations highlight the capability of the sensor to operate inside a soft actuator and validate the proposed rigid body transformations (P_robot_ to P_cap_) to extend the reach of the shape sensor. While the half circle channel in the actuator cap serves as a method to register the FOSS orientation and location relative to the actuator, this could be an issue for smaller actuators, given the minimum bend radius (10 mm) of the sensor. This is an application area that requires further development. For larger soft material robotic structures, we expect this to be less of an issue, and we suspect that the rigid registration feature could be integrated fully into larger structures if required.

In our contact detection, planar mapping, and material stiffness experiment, we demonstrated the capability of the actuator-sensor combination to detect a variety of features in its environment. One notable feature of the actuator-sensor combination is that its passive compliance enables it to deform on contact with external objects or surfaces. Since these deformations can be detected with submillimeter accuracy with the FOSS, contact detection and planar mapping become relatively straightforward tasks. Furthermore, since the FOSS performance is not impeded during actuation, the actuator-sensor combination has demonstrated the capability of measuring the relative stiffness of soft materials.

While the FOSS has proven to be a very effective sensing platform, a limitation for some applications will be data processing. In this evaluation platform, each data packet contained over 1700 points of position data across the length of the fiber (see Supplementary Data for more information). While these are more data than necessary for most scenarios, such as continuum arm applications,^34^ more advanced data collection methods need to be explored to downsample the data to reduce the computational overhead. However, akin to finite element analysis, where a coarse or fine mesh can be applied depending on the level of precision required, the FOSS enables similar capabilities where deformations or deflections (such as those from a collision or contact with an obstacle) can be evaluated with greater precision as needed.

A final consideration worth highlighting is the relative ease with which the FOSS can be integrated into a range of materials and robotic systems. The furcation tubing serves as a very flexible, protective housing that enables the sensor to be integrated into textiles, continuum-type arms, compliant mechanisms, and even rigid-robotic systems.^35^ This is an important distinction to make about this FOSS and soft material robotics: they do not need to draw from the same material library. This can be an advantage or disadvantage depending on the application. An advantage worth noting concerns one of the challenges of soft material systems, in which large deformations can rapidly degrade the useful life of the material through fatigue. When soft material sensor solutions are integrated into soft material actuators using the same material library, the fatigue life and hysteretic properties of these materials can pose challenges with calibration and measuring changes in system performance over time. We hypothesize that by relying on the soft material sensors alone as a state feedback sensor, it may be difficult to determine which system is degrading faster—the sensor, the actuator, or both at the same rate. With the proposed FOSS, we hypothesize that the easily verifiable precision of the sensor will enable more reliable state feedback control, as well as material health monitoring capabilities such as the ability to detect when a soft actuator may need to be repaired or replaced.

## Conclusions and Future Work

We presented new advancements in the fabrication and characterization of soft actuators with an integrated FOSS. More specifically, we described enhancements to a multistep, soft actuator molding process that enables the integration of a FOSS into the body of a soft material actuator. We also described a process of registering the tip position of an actuator with a custom designed actuator cap and a method for extending the spatial reach of the soft actuator. Finally, through a variety of experiments—tip twist, linear translation, bending, collision location detection, planar mapping, and material stiffness detection—we demonstrated the capability of the FOSS to accurately detect the shape and tip position of the soft actuator. Although the experimental data presented in this study focus primarily on planar, open loop shape sensing of a fiber-reinforced bending actuator, we intend for this soft actuator-sensor combination to provide a platform for advanced studies of the role of dynamics and control of soft robotic systems, ranging from finger sized to human sized.

## Supplementary Material

Supplemental data

Supplemental data

## Notes

^†^ Soft Robotics, Inc.

^‡^ “Big Hero 6,” Walt Disney animated film, 2014.
