# Supplementary material for: Fiber Optic Shape Sensing for Soft Robotics
Source: Soft Robot. 2019 Oct 4;6(5):671–84. doi: 10.1089/soro.2018.0131 (PMC6786339; doi:10.1089/soro.2018.0131)
Supplement: Supplemental data [file Supp_Data.pdf]

Supplementary Data

In the following Supplementary section, we present an overview of the shape data collected with fiber optic shape sensing (FOSS) by Luna Innovations, Inc. (Blacksburg, VA). In many ways, the shape sensor data are similar to the position data collected from a motion capture system, where multiple cameras calculate the three-dimensional positions of specialized markers. At each time step, a motion capture system records the X, Y, Z position of a marker as long as it's within the field of view of the cameras. For example, if a marker's position was sampled for 1 s at 100 Hz there would be 100 X, Y, Z data points. With the FOSS, the operator can choose to collect much more data. In this study, there are 1729 X, Y, Z position data points that describe the shape of the 1.35 m long sensor at each time step. Therefore, sampling the shape of the sensor for 1 s at 100 Hz produces 1729 times more data points compared to the motion capture example. More specifically, the shape data are a structure format. The size is $1 \times N$, where N indicates the number of scans. Inside each field of the structure data, there are 1729×3 data, where three represents the X, Y, Z position of each index (1–1729) relative to the FOSS origin (Fig. 5). Supplementary Table S1 presents a downsampled example where the X, Y, Z position data are presented at every 100th index. This rich data set enables the identification of expected geometry such as the semicircular slot in the cap which in turn can be analyzed to determine the index of actuator tip position, the cap orientation, or manipulated through rigid body transformations to extend the reach of the sensor as described in Equations (1–4).
