# Supplementary material for: Fiber Optic Shape Sensing for Soft Robotics
Source: Soft Robot. 2019 Oct 4;6(5):671–84. doi: 10.1089/soro.2018.0131 (PMC6786339; doi:10.1089/soro.2018.0131)
Supplement: Supplemental data [file Supp_Table1.docx]

Supplementary Table S1. Downsampled Example of the X, Y, Z Position Data from the Fiber Optic Shape Sensing at Every 100th Index Up to 1700 for a Single Time Step

**
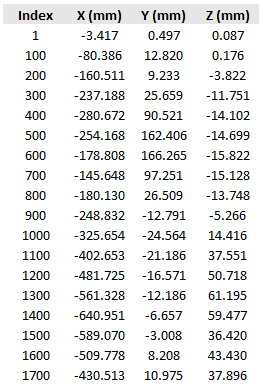
**
